# Oligodendroglia heterogeneity in the human central nervous system

**DOI:** 10.1007/s00401-021-02390-4

**Published:** 2021-12-03

**Authors:** Luise A. Seeker, Anna Williams

**Affiliations:** Centre for Regenerative Medicine, Institute for Regeneration and Repair, Edinburgh BioQuarter, University of Edinburgh, Edinburgh, UK

**Keywords:** Oligodendroglia, Oligodendrocytes, Heterogeneity, Myelin

## Abstract

It is the centenary of the discovery of oligodendrocytes and we are increasingly aware of their importance in the functioning of the brain in development, adult learning, normal ageing and in disease across the life course, even in those diseases classically thought of as neuronal. This has sparked more interest in oligodendroglia for potential therapeutics for many neurodegenerative/neurodevelopmental diseases due to their more tractable nature as a renewable cell in the central nervous system. However, oligodendroglia are not all the same. Even from the first description, differences in morphology were described between the cells. With advancing techniques to describe these differences in human tissue, the complexity of oligodendroglia is being discovered, indicating apparent functional differences which may be of critical importance in determining vulnerability and response to disease, and targeting of potential therapeutics. It is timely to review the progress we have made in discovering and understanding oligodendroglial heterogeneity in health and neuropathology.

## Introduction

To state the obvious, humans are not the same as mice, rats, zebrafish or other preclinical model animals. Although all our cells are different, the reason that we are human is due to our brain structure, and so it could be argued that understanding the physiological heterogeneity, pattern and biology of human brain cells may be even more important than with other cells. This difference and insufficient understanding of human brain cells may be reflected in the almost complete lack of effective treatments for neurodegenerative diseases. While initially research on the human central nervous system (CNS) has focussed for decades on neurons, more recent developments in the field show the important role of glia—that were first only seen as sticky substance that held the neurons in place [115]—in health and disease. Here, we will focus on oligodendroglia, the most prevalent cell lineage within the white matter, which include oligodendrocyte precursor cells (OPCs), immature pre-myelinating oligodendrocytes and mature myelinating oligodendrocytes. Mature oligodendrocytes form membrane extensions which wrap around nerve axons, forming the myelin sheath. The myelin sheath allows fast conduction velocity of electrical impulses along axons and provides metabolic support from the oligodendrocyte cell body to the underlying axon [34, 68]. Until relatively recently, we have tended to consider oligodendrocytes as one cell type, as they all form myelin, however, there is increasing evidence that oligodendroglia are morphologically [87] and functionally heterogeneous [55] depending on their developmental origin [17, 21, 100], differentiation stage, regional [87, 114] and anatomical location [30, 45, 74, 87], sex [19, 117], age [25, 69, 96, 112, 117] and transcriptome [55] and that this pattern of heterogeneity changes in disease [55].

This year is the centenary of the publication of the first description of oligodendroglia, beautifully drawn by Del Río Hortega, after using his newly developed silver carbonate procedure to stain cells in 1921 [94]. He named these cells oligodendroglia as glial cells with few processes (Greek: oligos = “few”, dendron = “tree”, or more freely “process”) and he described finding them throughout the CNS in grey and white matter [94]. The existence of these cells was doubted initially, but accepted in 1924, with support also from the American-Canadian neurosurgeon Wilder Penfield [86]. In 1928, Del Rio Hortega published a comprehensive overview of oligodendroglia, describing their heterogeneity in terms of their morphology and he speculated that they were involved in the formation of myelin, similarly to the Schwann cells of the peripheral nervous system [95]. One hundred years on, it seems timely to review where we have reached in terms of understanding different types of oligodendroglial heterogeneity, how it occurs, and what it means for CNS health and disease, across the human life course. We will focus on humans where possible, as rodents, used in preclinical models have relatively little white matter in comparison to brain volume [106, 127], and so may not well reflect human physiology. When considering disease, we will also focus mostly on oligodendrocyte heterogeneity in the classical CNS demyelinating disease of adulthood—multiple sclerosis (MS). Understanding human oligodendroglial functional heterogeneity may help us understand CNS health and disease, leading to improved therapeutic strategies.

## What is heterogeneity?

**Fig. 1:**
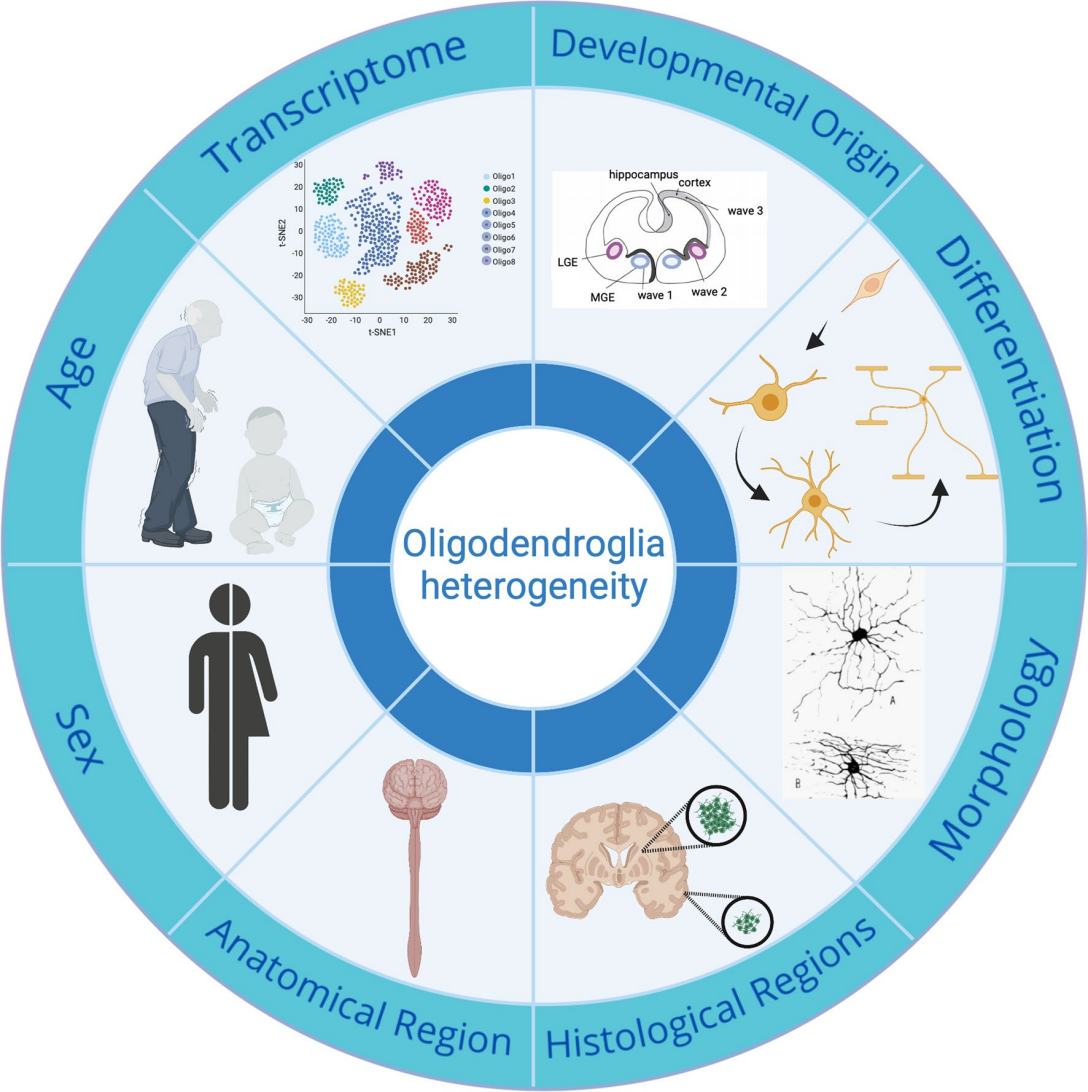
Summary of sources of oligodendroglia heterogeneity. Created with BioRender.com

The word heterogeneity is derived from the Greek ‘heteros’ meaning different and ‘genos’ meaning ‘kind’ and so can be defined by diversity in state or content, which we will interpret here as any differences in distinguishing oligodendroglia from each other. According to this definition, oligodendroglia are heterogeneous in their developmental origin [17, 21, 100], morphology [87] and capacity to myelinate [21] depending on their regional niche (grey matter vs. white matter) [87, 114], anatomical site [30, 45, 74, 87], donor sex [19, 117] and donor age [25, 69, 96, 112, 117] (Fig. 1). These differences may be intrinsic [7] and/or extrinsic with cues from the tissue environment [36, 71, 83, 114], including the calibre of the axon being myelinated [7, 52, 80]. We will address these in turn.

### Oligodendroglial heterogeneity as defined by developmental source

In development, OPCs of the cerebral hemispheres, cerebellum and spinal cord are derived from neural stem cells (NSCs) at different locations. In mouse forebrain development, OPCs are derived from NSCs in three waves. The first wave of OPCs originate from the subventricular zone (SVZ) from the medial ganglionic eminence and the anterior entopeduncular area at embryonic day E12.5 [61]. However, in normality, these are completely replaced by a second wave of OPCs from the lateral and caudal ganglionic eminences by the age of E15.5 and a third wave from the dorsal SVZ into the cortex [61, 101]. Cerebellar OPCs have been shown to mostly originate from extracerebellar brain regions [39], migrating from the metencephalic ventral rhombomere 1 region arising at E11.5 and arriving E16.5–E18.5, proliferating further and being added to by OPCs from the cerebellar ventricular zone [42] and after birth by OPCs from the fourth ventricle [92, 128]. In the spinal cord, the first OPCs arise after E12.5 from the ventrally located premotor neuron domain [88, 93, 118] and later (E15.5) from more dorsal precursor domains of the spinal cord [17, 31], but by birth, these dorsally derived OPCs make up only 10–20% of the OPCs [31, 113]. These developmental OPC generation routes appear conserved in humans [57, 58, 78, 90], but there may also be an additional route as primates show an enlarged outer ventricular zone during development compared to rodents [29, 111, 125] and progenitor cells in this zone produce not only neurons [9, 70] but also OPCs [49]. Furthermore, a recent study has shown the presence of OPCs in human embryos at post conception week 8–11 which is earlier than previously assumed and which supports the notion that human oligodendroglia develop in several waves in parallel to what is known in mice [14].

It is not fully understood if this regional difference in origin translates into biological differences in later oligodendroglial function, and if these oligodendroglia are intrinsically different or influenced by the local environment. The transcription factors expressed by the three waves of OPCs in the mouse forebrain are different, with ventral, medial and dorsal wave OPCs expressing Nkx2.1, Gsh32 and Emx1, respectively [61], suggesting intrinsic differences that may lead to different functions. Furthermore, dorsally derived mouse spinal cord OPCs show enhanced proliferation, recruitment and differentiation into remyelinating oligodendrocytes in response to demyelinating injury in vivo compared to ventrally derived OPCs, although this difference declines with age [21, 130]. The oligodendrocytes subsequently differentiating from these spinal cord OPCs also appear intrinsically different as cultured rodent spinal cord oligodendrocytes produce myelin sheaths that are longer and thicker than forebrain-derived oligodendrocytes even when wrapping the same diameter polymer fibres [7]. However, oligodendrocytes from the same origin but residing in white or grey matter also behave differently [26, 105], suggesting that the local environment where oligodendroglia reside also changes their function. We will return to heterogeneity related to location below, but this evidence suggests that developmental oligodendroglial heterogeneity depends on a combination of regional intrinsic and environmental influences, and that there may be cellular expression markers to define this.

### Differentiation markers of oligodendroglial heterogeneity

**Fig. 2:**
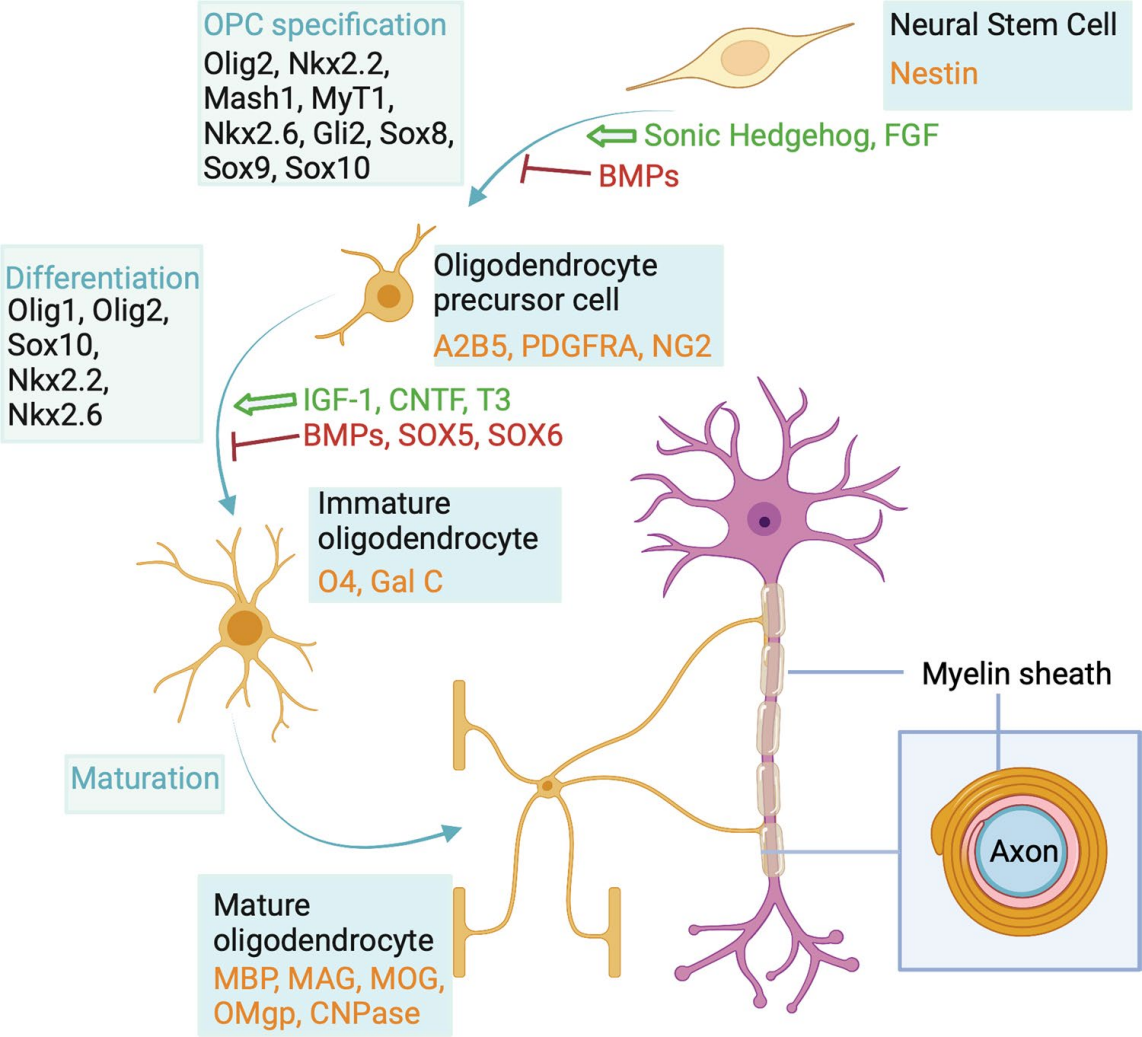
Common markers used for differentiation stages of the oligodendroglia lineage. Created with BioRender.com

**Fig. 3:**
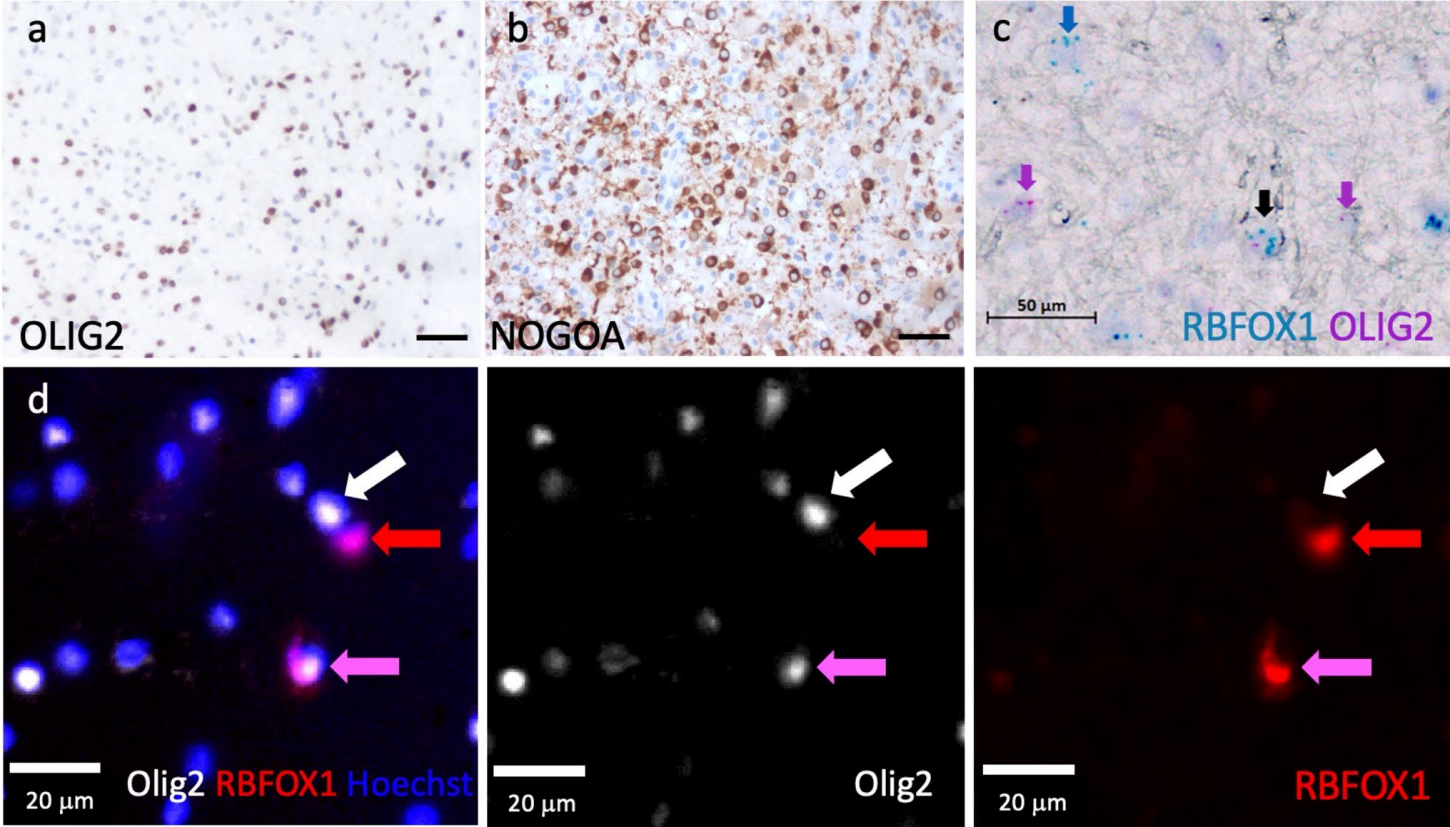
Examples of molecular markers of oligodendroglia on human postmortem brain tissue. **a** Colorimetric immunohistochemistry using an antibody against OLIG2. **b** Colorimetric immunohistochemistry using an antibody against NOGOA. **c** In situ hybridisation (BaseScope Duplex) using probes against RBFOX1 and OLIG2 demonstrating heterogeneity. The pink arrows mark OLIG2-positive oligodendroglia that are RBFOX1 negative, the blue arrow indicates a cell that is RBFOX1 positive, but OLIG2 negative (not oligodendroglial) and the black arrow a double positive cell. **d** Immunofluorescence using antibodies against OLIG2, RBFOX1 and the nuclear marker Hoechst, again demonstrating heterogeneity. The white arrow marks a cell with only a OLIG2 marker, the red arrow a cell with only a RBFOX1 marker (not oligodendroglial) and the pink arrow an OLIG2+ cell also expressing RBFOX1

The advent of immunohistochemistry has allowed us to use protein molecular markers to distinguish oligodendroglia from the different developmental origins and also different stages in the differentiation of oligodendroglia in development and adulthood (Fig. 2). This has enabled us to understand stages in the continuum of oligodendroglial differentiation from oligodendrocyte precursor cells (OPCs) to immature oligodendrocytes and then into mature myelinating oligodendrocytes. The markers commonly used to distinguish these are illustrated in Figs. 2 and 3, and will just be stated here as they have been reviewed extensively [64, 77]. These are perhaps more described and used in the in vitro cell culture setting and in development rather than in tissue in adult human CNS, except in the context of a response to pathology where the developmental oligodendroglial differentiation pathway is thought to be at least partly recapitulated in the regenerative response. In preclinical models, or even human stem cell-derived oligodendrocytes cultured in vitro, it is possible to add reporters to reflect the different stages along the differentiation pathway in real time and live. This is particularly effective in the embryonic zebrafish where it is relatively straightforward to use multiple different fluorescent reporters in live fish imaged over time to see the differentiation process in vivo. Live imaging of cortical oligodendroglia with fluorescent reporters is also possible through skull windows in mice, but imaging deeper white matter oligodendroglia is more difficult. These two methods have revealed a heterogeneity of oligodendroglial responses to damage, with remyelinating oligodendrocytes able to be derived from OPCs (as in development) but also surviving oligodendrocytes can extend new processes for myelin repair [6], though perhaps less successfully [82]. There is evidence suggesting this may be similar in humans, as radionucleotide labelling in humans post atomic bomb testing shows that remyelinated lesions in MS brain contain oligodendrocytes which still contain high levels of the radionucleotide, suggesting they are old, rather than from proliferative OPCs which will have diluted out this label [123]. Further evidence from human biopsies of acute and inflammatory MS lesions shows that, unexpectedly, there is not an increase in OPCs or immature oligodendroglia in such lesions, suggesting that these may remyelinate using the oligodendrocytes that are already there [44]. Finally, the mistargeting of myelin to neuronal cell bodies that is seen in zebrafish oligodendrocytes that survive and remyelinate is also found in human MS tissue in the peri-demyelinated lesion areas [82]. These live-imaging data from other species have provided us with information that it is impossible to obtain from humans and means that we need to think again about the heterogeneity of responses of human oligodendrocytes to pathology.

As well as using protein markers, we can use RNA in situ hybridisation probes to detect RNA markers of oligodendroglia. Improved technology using multiple short probes to different neighbouring parts of the same RNA, instead of full-length probes, with highly efficient signal amplification, has allowed specific and sensitive detection of single RNA molecules in human tissue—both fresh–frozen and paraffin [27] (Fig. 3). This technology also now allows detection of different splice variants, single base mutations and microRNAs or long non-coding (lnc) RNAs. The latter is of particular importance to oligodendroglial heterogeneity as some are selectively expressed in subsets of oligodendrocytes [55], and there is increasing interest in lncRNAs in driving oligodendroglial differentiation in health and regeneration in disease [43, 60, 119].

These molecular markers alter over differentiation in development and disease, along with well-described morphological changes from bipolar OPCs to highly branched complex oligodendrocytes.

### Morphological heterogeneity

In 1928, Del Rio Hortega published his comprehensive review of oligodendrocytes in the CNS, using his new Golgi–Hortega technique initially describing three types of cells on the basis of where they were found: interfascicular (cells aligned in rows along axonal tracts); perineuronal (next to neuronal cell bodies) and perivascular (around blood vessels). In addition, he then classified them on their morphology, dividing them into 4 types: Type I with small rounded cell bodies and many fine processes emerging radially towards thinly myelinated axons, Type II with polygonal cell bodies, fewer and thicker processes directed to axons longitudinally, Type III with a large soma and one to four processes directed towards axons and Type IV with elongated cell bodies with one or two processes [77, 84]. This allowed him to speculate that oligodendrocytes were involved in the formation of myelin, similarly to the Schwann cells of the peripheral nervous system and emphasises how detailed descriptions of anatomy pave the way to more understanding. Although these categories are no longer used, in favour of molecular markers, again live-imaging work from the embryonic zebrafish using oligodendroglial reporters shows heterogeneous morphology in OPCs with functional differences by transcriptomic analysis [73].

### Subregional location determining heterogeneity

Del Rio Hortega also observed that his four morphological types were differentially distributed across CNS tissue, suggesting that grey and white matter OPCs may give rise to oligodendrocytes of different morphology, e.g. Type II oligodendrocytes were described as white matter specific [87, 94]. Although there is a greater oligodendroglia density in the white matter, a proportion of them also resides in the grey matter and their behaviour at these different locations differs, at least in the mouse. Mouse white matter OPCs have been shown to proliferate more readily when stimulated [46] and produce more mature oligodendrocytes [114] than mouse grey matter OPCs. This difference is due to both intrinsic as well as extrinsic cues, because when white matter OPCs were transplanted into grey matter, they divided and differentiated more efficiently than grey matter OPCs, but not as efficiently as in the white matter environment [114]. As these white matter and grey matter OPCs were derived from the same source of progenitor cells, the observed difference may be due to environmental cues at key steps of the differentiation with a prolonged effect on the cells.

### Anatomical location determining heterogeneity

As well as grey/white matter differences, Del Rio Hortega described morphological oligodendrocyte differences with anatomical region, for example describing oligodendrocyte type IV as only present in the brain stem and in the spinal cord [87, 95]. It has been long known that spinal cord oligodendrocytes produce longer and thicker myelin sheaths than those of other CNS regions [45], but it remained unclear if this was due to intrinsic differences related to their different developmental origin, or secondary to environmental cues such as axonal thickness. Axons in the spinal cord have larger diameters on average than those in the brain and axonal thickness positively correlates with myelin thickness [7, 52, 80]. One study dissected the intrinsic and extrinsic components of this finding by culturing murine oligodendrocytes differentiated from spinal cord or cortical OPCs on synthetic polymer fibres [7]. This study showed that myelin sheath length was associated with both the origin of the cells and the diameter of the fibres. This indicated that oligodendroglia of different anatomical sites are intrinsically different but they can still recognise and adapt to different environmental situations. These functional differences may be reflected in the transcriptional signatures of oligodendroglia as in mice different subtypes of oligodendrocytes as defined by distinct marker gene expressions were found in brain compared to spinal cord, but also within different regions of the spinal cord. However, using lineage tracing, these oligodendrocyte subtypes did not seem to derive from OPCs from the different developmental origins [30], suggesting that even apparent intrinsic differences may not be due to the developmental origin but to early environmental cues during differentiation, at least in mice. This multiplicity of OPC developmental types, but convergence of these into all differentiating into the same subtypes of oligodendrocytes begs the question of why this system has evolved. One can speculate that the OPCs have other functions e.g. in axonal pruning [15, 122], unrelated to the oligodendrocyte differentiation, or that we still do not understand the intricacies of oligodendrocyte heterogeneity at high enough resolution.

### Heterogeneity related to sex

As well as regional differences in oligodendroglia, are there fundamental differences between female and male oligodendroglia? These may be determined by their sex chromosome content, or by influences of the sex steroid hormones. Oligodendroglia are influenced by estrogens, progestogens and androgens. Estrogens have been implicated in directly promoting neural stem cell and OPC proliferation, differentiation and myelination from in vitro experiments, as well as ameliorating responses to neuroinflammatory conditions such as Experimental Allergic Encephalomyelitis (EAE) in preclinical models and MS in humans [4, 72] (reviewed in [62]). However, the latter effect directly on oligodendroglia in vivo is difficult to discern from an indirect effect on the immune response and on other cells. Progesterone has similarly been positively implicated in enhancing oligodendrocyte formation and myelination [35, 37]. Testosterone has also been shown to enhance remyelination in male mice [51], especially compared to castrated male mice [10], at least in part due to a direct effect on oligodendroglia. This androgen response may also act through signalling through the androgen receptor on microglia and astrocytes with contribution from the hedgehog pathway, and with a secondary effect on remyelination [66]. In developmental myelination, oligodendroglia will be exposed to maternal sex hormones, and oligodendroglia involved in myelin maintenance and remyelination are exposed to the sex hormones of the adult. In MS, although fewer males are diagnosed with the disease, their prognosis is worse, and remyelination may be more extensive in early MS postmortem lesions in females than in males [63]. There is also emerging exciting evidence that gonosomal genes may directly influence brain cell function in neurodegenerative diseases [16, 23, 67] rather than simply through sex hormone differences. In a genetically modified Alzheimer’s disease mouse model, the expression of the X-linked gene Kdm6a, one of the few genes still expressed on the otherwise inactivated X-chromosome, was associated with longer survival in both sexes [23]. In addition, the inhibition of the Y-chromosomal gene SRY in a murine Parkinson’s disease model reduced clinical signs and improved dopaminergic neuron survival [67]. However, the effect of gonosomal genes on oligodendroglia function remains to be explored and little is known specifically about sex-related differences in oligodendroglia in humans and the contribution to function in health and disease.

### Heterogeneity related to age

Over the human lifespan, oligodendroglia can form and maintain myelin. The first OPCs emerge in the developing human brain at 10 weeks’ gestation, followed by an expansion of the population (15–20 weeks) [57, 58, 97] and myelination commences at 30 weeks’ gestation, but most occurs postnatally [50, 53, 106]. This progresses in a rostral to caudal manner, with spinal cord and cerebellum myelination before the cerebral hemispheres, finishing with the prefrontal cortex [53, 57, 58]. C14 Carbon dating in humans suggests that oligodendrocyte numbers are stable after 5 years of age [124], with slow myelin turnover, although there is evidence from rodents [103, 110, 121] and humans [104], that neuronal activity can drive new myelination, for example by exercise and learning new tasks. With ageing, there is reduced myelin integrity [8], occurring initially in areas of the brain that were myelinated first, as seen on micro-structural magnetic resonance imaging (MRI) [40], and at a pathological level, there is detachment of the paranodal loops at the ends of the myelin sheaths [48, 107], suggesting poorer oligodendroglial function.

This heterogeneity in oligodendroglia/myelin over the normal life course may be important in understanding their vulnerability and response to diseases that occur at different ages. The developmental timing of OPC emergence and myelination make oligodendroglia vulnerable to perinatal hypoxic and inflammatory injury, for example due to premature birth. This reduces the maturation of immature oligodendrocytes into myelinating oligodendrocytes, leading to white matter injury and can result in cognitive and/or physical developmental defects [116]. Genetic diseases causing oligodendrocyte defects can cause dysmyelination (such as in Pelizaeus–Merzbacher disease [84]) which usually leads to early death.

In early to mid-adulthood, the commonest cause of non-traumatic neurological disability in the ‘first world’ is MS, where focal areas of demyelination occur in the CNS with oligodendrocyte loss, and attempts at remyelination (reviewed in [32]). Furthermore, in older age, cerebral small vessel disease is very common [108] and is characterised by white matter changes on magnetic resonance brain scans which correlate with clinical cognitive impairment [24] and endothelial cell, oligodendroglial and myelin changes by pathology [89]. Oligodendrocyte pathology has also been increasingly implicated in other neurodegenerative diseases usually associated with ageing that were previously thought to be classically neuronal, such as progressive supranuclear palsy [2, 99], Huntington’s Disease [109] and Parkinson’s disease [1], as well as psychiatric diseases such as major depressive disorder [81].

These changes suggest that human oligodendroglia change with age, but it is uncertain whether different subsets of these are more prominent in development, adult myelin maintenance, and ageing, whether these confer differential susceptibility to pathology or therapeutic responses to, for example, pro-remyelination drugs. To try and understand these different nuances in oligodendroglial behaviour over age and in pathology, researchers have turned to assessing transcriptional heterogeneity, particularly at a single cell level.

### Transcriptional heterogeneity

Recent technology has allowed us to examine heterogeneity within oligodendroglia at an unprecedented level by examining transcripts from single cell RNA sequencing (scRNAseq) or single nuclear RNA sequencing (snRNAseq), allowing phenotyping cells using multiple gene transcripts as a readout of their function. This is now possible at scale, allowing classification of oligodendroglia using similarities (and differences) between these transcripts, for tens to hundreds of thousands of cells, limited currently only by both cost and computational power for the analysis, albeit with, at least with droplet technology, capture of considerably less than half of the expressed transcripts [129]. There has been an explosion of information using these tools about many different cells from many different tissues of a variety of species, all showing that the broad groupings of cells that we have worked with for years contain disparate cells. This is being at least in part driven by an international initiative to sequence all of the different cells of the human body and have the data as open access within the Human Cell Atlas [91] (www.humancellatlas.org).

First scRNAseq studies in adult and juvenile mouse CNS samples found distinct oligodendroglia states and markers for differentiation stages [74, 126]. The oligodendroglia states varied with tissue region indicating regional heterogeneity. At the same time, a proof of principle scRNAseq study showed that similar investigations are possible in adult and foetal human CNS [22]. As the technology improved, snRNAseq allowed use of archived human post-mortem fresh–frozen tissue, significantly increasing the breadth of tissue analysed [41, 65]. Both mouse and human studies show that all expected cell types can be identified using canonical marker genes and that those cell types can typically be further subclustered into groups of cells that express similar genes and differ from other subclusters in their gene expression which also indicates a distinction of differentiation stages and cellular function [55].

In humans, axons are mostly myelinated post-partum and therefore human foetuses lack proper myelinated white matter. However, scRNAseq has shown that human foetal oligodendroglia express EGFR from 20 gestational weeks onwards and include pre-OPCs, OPCs, and SPARCL1-positive OPCs that have transcriptional similarities with the astrocyte lineage [33]. Different to murine OPCs, where typically one daughter cell continues to divide and produce more OPCs whereas the other daughter cell differentiates, in human foetuses, OPCs divide symmetrically several times before they start differentiating [49]. This may be one of the mechanisms that leads to a ~ 3000 times greater relative white matter in humans compared to rodent animal models. The expression of PCDH15 in OPCs leads to daughter cell repulsion which is likely associated with new OPCs infiltrating other brain regions [49], but also allows further proliferation which is inhibited by high OPC density [49].

Although the myelin transcriptome/proteome of mice and humans overlap, these developmental differences, and RNAseq and proteomic studies have shown that there are distinct components present only in one or the other species [54]. In addition, animals are incomplete preclinical models for MS, with a lack of description of MS in any animal species including non-human primates except perhaps in a population of Japanese macaques with an endemic herpesvirus infection, where a low percentage of animals each year develops an MS-like disease [5, 38]. This underlines the importance to better understand human oligodendroglia using human tissue and cells.

Sc/snRNAseq studies in human adults mostly focus on postmortem tissue, simply due to the pragmatics of obtaining samples, comparing oligodendroglial subgroups between mouse and human, and between human health and different neurological diseases.

**Fig. 4:**
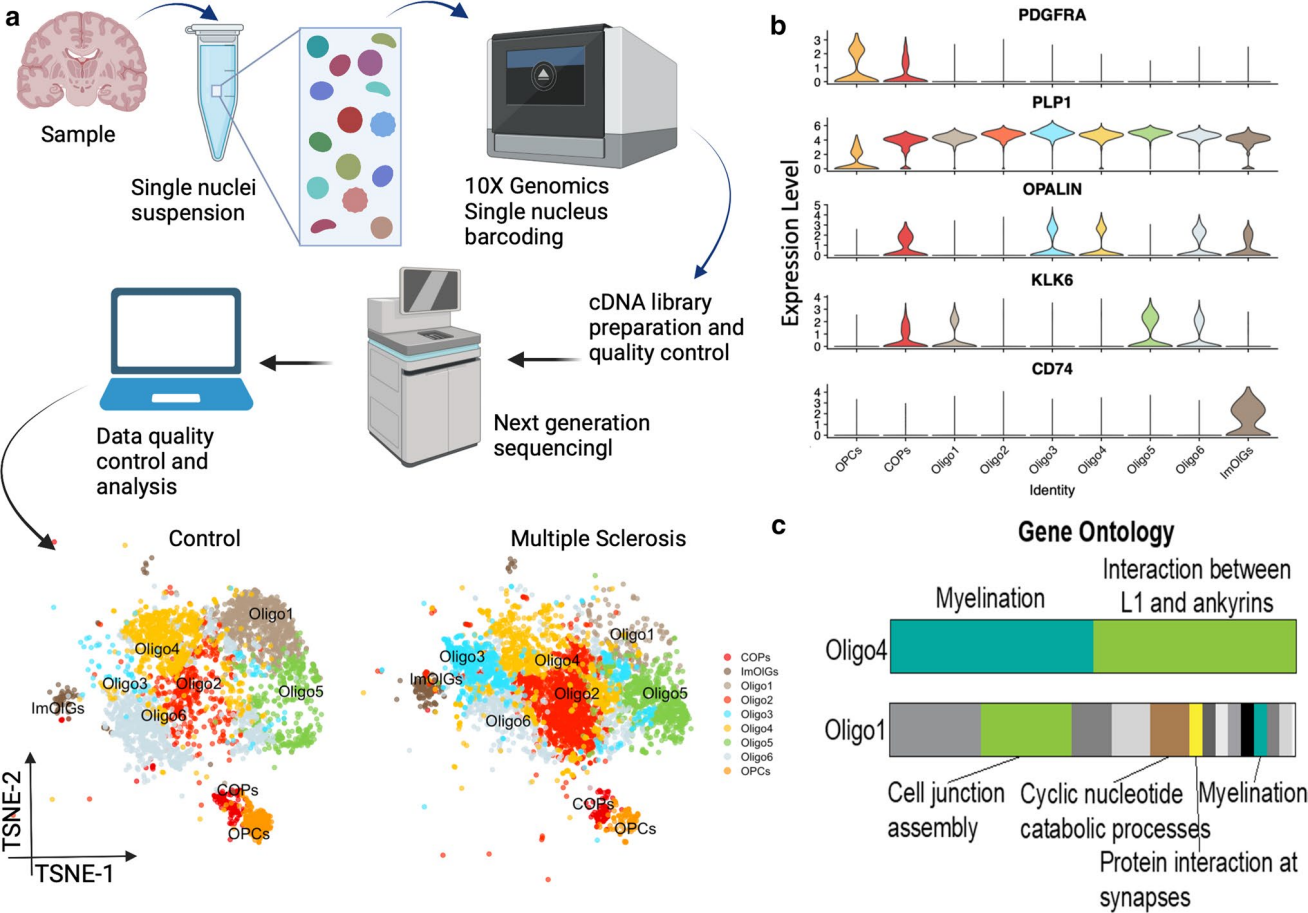
Oligodendrocyte heterogeneity at the RNA level. **a** Simplified standard workflow of an snRNAseq experiment. Shown data are adapted from Jäkel & Agirre et al. [55] and shows postmortem human brain affected/unaffected by multiple sclerosis. **b** A selection of marker genes visualises transcriptional differences between oligodendrocyte states. **c** Gene ontology analysis of differentially expressed genes in Oligo4 and Oligo1 indicates functional differences in oligodendroglia states. Figure (**c**) kindly provided by Sarah Jäkel. Created with BioRender.com

Using human postmortem brain tissue from the MRC Edinburgh Sudden Death Brain Bank, with pathologically normal human brain, and from the MS Society UK MS tissue bank with pathologically proven MS, we performed snRNAseq using 10 × technology on 20 different white matter brain samples from 4 MS patients and 5 controls [55]. Using this dataset, we subdivided our oligodendroglia into 8 subclusters, including OPCs, committed oligodendrocyte precursors, and 6 types of oligodendrocytes all expressing myelin protein transcripts such as MBP and MOG, but with other transcriptional differences [55]. These subclusters are identifiable by single or groups of transcript markers, some of which are defined in Fig. 4b. We identified that in MS brain, there are fewer OPCs and a shift in the proportions of oligodendrocytes in these subclusters, showing that MS pathology leads to changes in oligodendrocyte functional heterogeneity with some clusters over-represented and some under-represented (Fig. 4a). But what do these differences in subclusters mean functionally, and how does the shift in oligodendroglial composition affect the biology/pathology in MS? We are not yet certain, as we inevitably only have one timepoint in postmortem human data, but examination of the gene transcripts allows us to speculate, generate hypotheses and in the future test these in other systems. Comparison of the RNA transcripts using gene ontology analysis suggests that over half of the transcripts in Oligo4 cluster cells relate to myelination—more than with any of the other subclusters, and suggesting that these cells are been driven to try to actively myelinate/remyelinate. This cluster is under-represented in MS tissue, allowing the hypotheses that either these are selectively vulnerable in MS, or that these cells fail to regenerate after global loss of oligodendrocytes in MS. It is tempting to speculate that these oligodendrocytes are the ones that might be the most useful to generate for successful remyelination in MS. Conversely, Oligo1 subcluster cells have relatively few RNAs that correspond to myelin genes, and instead express transcripts more related to signalling and metabolism. This leads to an interpretation that these cells have completed active myelin formation and are simply maintaining their sheaths, and instead are focussing on signalling and metabolic support to the underlying axon—an essential role for maintenance of myelin throughout life. These are also under-represented in MS tissue, suggesting that few oligodendrocytes even if they achieve remyelination, manage to then provide the same support as in normality. Instead, the subclusters that are over-represented in MS are ones that we believe to be more immature, with markers such as OPALIN, and perhaps represent the block in oligodendrocyte formation which has been much described in the literature [32]. Another cluster over-represented in MS brain, called ‘immune-oligos,’ expresses major histocompatibility complex II (MHC-II) genes, confirmed at the protein level, similarly to in EAE, a mouse T cell-driven model of MS [28]. This may suggest that in MS, some oligodendrocytes are capable of presenting antigen, which may contribute to the immune-driven MS pathology. Whether the oligodendroglia in these different clusters are fixed in these states in adult tissue, or can transition to and from other functional states is also not known, and will only be resolved by functional modelling of live cells, as RNAseq simply gives us a snapshot of the function of each cell at the time of freezing the tissue. If fixed, then clusters represent co-existing distinct oligodendrocyte morphologies, with static presumed functional and regional differences, perhaps suggesting a division of labour for these cells, or that certain oligodendrocytes myelinate certain neuronal axonal types, etc. However, if, in adulthood, they are dynamic and oligodendrocytes can change gene expression signatures, this suggests possibilities for adaptation to the tissue environment over time and potentially to pathology. Bioinformatically, trajectory inference methods have been developed to predict how cells differentiate from immature to mature cells, ordering them based on their transcriptional similarity. However, these methods are not designed to identify transitions in adult tissue at one timepoint, where transitions may be more stochastic and in multiple directions between clusters.

The next question is whether this skewed oligodendroglial heterogeneity in MS is MS-specific or a more general response to pathology. This is not yet fully resolved, but in a similar study, using tissue from donors with major depressive disease, by direct comparison of the datasets, the authors identified some similar oligodendroglial subclusters [81]. Many further sc/snRNAseq studies have identified altered human oligodendroglia heterogeneity with different diseases including further work in multiple sclerosis [102], Alzheimer’s disease [75, 79], Huntington’s disease [3], Parkinson’s disease [1], neuropsychiatric disorders [12, 13, 81], and glioblastoma [20]. In these studies, inevitably, various different numbers of oligodendrocyte and OPC clusters were identified, likely depending on probabilities and decisions about data pre-processing and resolutions in the analysis, as well as potential regional differences and differences in the proportions of grey and white matter. These latter possible differences have not yet been well explored in human tissue with these techniques. However, despite this, parallels across datasets are clear: OPALIN and RBFOX1 are clearly reported as markers for two different oligodendroglia states [55, 79]. In addition, both of those studies [55, 79] detected “Immuno-oligos” that express major histocompatibility complex class II genes suggesting antigen presentation capabilities, and these were enriched in both MS and Alzheimer’s disease compared to control groups [55, 79]. As the datasets increase in number and size, we will need a concerted response to integrate relevant datasets from the human white and grey matter, in health and across a variety of diseases, with a subsequent agreed nomenclature to allow us to improve our understanding of normal and pathological transcriptional distinctions among the oligodendroglia population.

These findings have increased interest in the oligodendroglial influence in classical neurodegenerative diseases, partly as therapeutic options are wider with these cells, which, unlike neurons can regenerate. This leads us back to our section on using markers to identify oligodendroglia, as this transcriptomic analysis gives us new markers of oligodendroglia, both at the broad level, e.g. OPCs and mature oligodendrocytes and at the subcluster level, allowing us to validate the presence/number of these oligodendroglia in the CNS in different regions, diseases, etc. without needing to perform RNAseq. Furthermore, there are now new and improved techniques to spatially identify several or multiple of these markers in combination with either RNA probes or antibodies on human postmortem tissue, both in frozen and formalin-fixed paraffin sections. For example, previous studies including ours [55, 79] have shown that a subset of oligodendrocytes expresses the RNA Binding Fox-1 Homolog 1 (RBFOX1) which can be visualised and quantified using in situ hybridisation or antibody-staining techniques (Fig. 3). To this, we can add the even more recent development of on-tissue RNA-sequencing, giving spatial information, with some techniques at single cell resolution covering all genes, single cell epigenomics (e.g. ATACseq), lipidomics and proteomics and it is a very fast-moving field (reviewed in [56]). One can see that the knowledge of (and data from) oligodendroglial heterogeneity will only expand, with the limit perhaps being the ability to analyse such data and cost.

## Can we reconcile these sources of heterogeneity?

Above we discussed eight different sources of oligodendrocyte heterogeneity. For clarity and convenience, each source was discussed separately, but these clearly correlate, interact or even perhaps cause one another. For example, in development, OPCs change in morphology from a bipolar cell to one with many processes wrapping axons and this is accompanied by a transcriptional switch with the expression of myelin genes, allowing the formation of the myelin sheath. Similarly, the different developmental origin for example of spinal cord OPCs may influence their regional heterogeneity, their transcriptional signatures and this may determine (at least in part) their ability to myelinate axons with longer and thicker myelin sheaths than brain ones [7].

The current development of spatial transcriptomics technologies to measure multiplexed gene expression in situ [47, 76, 98] will allow us to better correlate transcriptional variation, regionality, morphology and the interactions between heterogeneous oligodendroglia and other CNS cells, not just restricted to whether different oligodendrocyte ensheath different axons, but in their interactions with astrocytes, microglia, vascular cells, immune cells, each other and indeed with different subtypes of these. This is clearly essential for understanding normal and pathological brain function, and will involve complex data analysis in three dimensions, preferably over time. We are likely to increase the sources and complexity of oligodendroglia (and other cell) heterogeneity by including measures of chromatin accessibility, DNA methylation, spatial gene expression, single cell proteomics, and lipidomics and integration of these data modalities is going to be challenging but hopefully exciting. The first step here (with a focus on neurons) has very recently been published through the Brain Research through Advancing Innovative Neurotechnologies (BRAIN) Initiative Cell Census Network using multimodal and multispecies investigations of the primary motor cortex [18].

## Challenges and future in studying human oligodendroglia heterogeneity

The challenges of understanding oligodendroglial heterogeneity are large, but importantly, although -Omics *predict* function, we do not really know yet what impact these changes have on the actual biological function of the cell. For example, do some subclusters represent oligodendrocytes which are primed to remyelinate, others which are dying and yet others which are designed to provide metabolic support to underlying axons? There are hints of this from the bioinformatics, but this needs to be tested experimentally. In the context of MS, are there some individuals who have more of the oligodendrocytes ‘good’ at remyelination, with less chance of disease progression and vice versa, perhaps matching the ‘good’ and ‘bad’ remyelinators detected by Positron emission tomography (PET) scanning in MS [11]. This leaves us excited for the future of using such information to identify disease or disease progression/prognosis if we can convert these findings into markers measurable in the living person. Is there a generic ‘pathological’ oligodendrocyte and/or a ‘regenerative’ one across all diseases, or some diseases? Can we use drugs to transition oligodendrocytes from such a ‘pathological’ state to a ‘regenerative’ one as a new therapeutic strategy? These are not easy questions to answer, as human oligodendroglia reflecting adult in vivo biology are difficult to grow in culture. However, progress is being made with human stem cell-derived cells used to form cultured organoids [59, 85] and injected into immunocompromised mouse brain [120] showing more fidelity to adult human oligodendroglia in vivo.

## Conclusion

One hundred years after the first description of oligodendrocytes, we are now in the exciting position that we have discovered that oligodendroglia vary in their function, at least in part depending on origin, location, age, sex and pathology, suggesting that this may be driven by a combination of regional intrinsic and environmental influences. Oligodendroglia are also seeing a revived popularity, with oligodendroglial components now implicated in many neurodegenerative diseases, and due to their potential tractability to therapeutics. We predict that in the future, we will increasingly use markers of these different subtypes of oligodendroglia to predict disease course and stratify patients for therapies, but we have some way to go!
